# Morphological Brain Changes after Climbing to Extreme Altitudes—A Prospective Cohort Study

**DOI:** 10.1371/journal.pone.0141097

**Published:** 2015-10-28

**Authors:** Raimund Kottke, Jacqueline Pichler Hefti, Christian Rummel, Martinus Hauf, Urs Hefti, Tobias Michael Merz

**Affiliations:** 1 Institute for Diagnostic and Interventional Neuroradiology, University Hospital and University of Bern, 3010, Bern, Switzerland; 2 Department of Intensive Care Medicine, University Hospital and University of Bern, 3010, Bern, Switzerland; 3 Support Centre for Advanced Neuroimaging (SCAN), Institute for Diagnostic and Interventional Neuroradiology, University Hospital and University of Bern, 3010, Bern, Switzerland; 4 Swiss Sport Clinic, 3014, Bern, Switzerland; Hungarian Academy of Sciences, HUNGARY

## Abstract

**Background:**

Findings of cerebral cortical atrophy, white matter lesions and microhemorrhages have been reported in high-altitude climbers. The aim of this study was to evaluate structural cerebral changes in a large cohort of climbers after an ascent to extreme altitudes and to correlate these findings with the severity of hypoxia and neurological signs during the climb.

**Methods:**

Magnetic resonance imaging (MRI) studies were performed in 38 mountaineers before and after participating in a high altitude (7126m) climbing expedition. The imaging studies were assessed for occurrence of new WM hyperintensities and microhemorrhages. Changes of partial volume estimates of cerebrospinal fluid, grey matter, and white matter were evaluated by voxel-based morphometry. Arterial oxygen saturation and acute mountain sickness scores were recorded daily during the climb.

**Results:**

On post-expedition imaging no new white matter hyperintensities were observed. Compared to baseline testing, we observed a significant cerebrospinal fluid fraction increase (0.34% [95% CI 0.10–0.58], p = 0.006) and a white matter fraction reduction (-0.18% [95% CI -0.32–-0.04], p = 0.012), whereas the grey matter fraction remained stable (0.16% [95% CI -0.46–0.13], p = 0.278). Post-expedition imaging revealed new microhemorrhages in 3 of 15 climbers reaching an altitude of over 7000m. Affected climbers had significantly lower oxygen saturation values but not higher acute mountain sickness scores than climbers without microhemorrhages.

**Conclusions:**

A single sojourn to extreme altitudes is not associated with development of focal white matter hyperintensities and grey matter atrophy but leads to a decrease in brain white matter fraction. Microhemorrhages indicative of substantial blood-brain barrier disruption occur in a significant number of climbers attaining extreme altitudes.

## Introduction

Altitude related medical problems are gaining importance and attention as an increasing number of trekkers and recreational climbers attempt ascents to very high (3500m to 5500m) or extreme altitudes (>5500m) [1]. The possibility of long-term cerebral sequelae from exposure to severe hypobaric hypoxia has been a topic of controversy for decades [2–4]. Structural cerebral changes detected by magnetic resonance imaging (MRI) have been reported after high-altitude climbs [5–12]. These include findings of cortical atrophy and white matter hyperintensities in mountain climbers ascending to altitudes between 4810 m and 8848 m, the majority of which did not suffer from cerebral forms of high altitude illness, such as severe acute mountain sickness (AMS) or high altitude cerebral edema (HACE) [5, 7–10]. MRI studies of climbers after the occurrence of clinical overt HACE have shown reversible findings of vasogenic edema [11] and of microhemorrhages [6, 12], both with a predilection for the splenium of the corpus callosum. Microhemorrhages in the corpus callosum after high altitude exposure represent evidence for a disruption of the blood-brain barrier and have been postulated to be specific for HACE [12].

Published imaging studies in high altitude climbers represent case series or cohort studies in a small number of subjects and data on severity of hypoxia and signs and symptoms of high altitude illness was not prospectively collected. Often, imaging was obtained only after high-altitude exposure and non-climbers served as controls [6–8, 10, 11, 13]. The retrospective clinical diagnosis of cerebral forms of high altitude illness occurring in the context of challenging conditions during a high altitude climb can be difficult, even when applying recommended scoring systems [14, 15].

The aim of the study at hand is to evaluate the occurrence of structural cerebral changes in a large group of climbers by comparison of MRI studies before and after ascent to extreme altitude and to correlate these findings with prospectively collected data on severity of hypoxia and signs and symptoms of cerebral forms of high altitude illness during the climb. Based on the results of previous studies, we hypothesized that structural cerebral changes such as cortical atrophy and white matter hyperintensities would occur more frequently in the most hypoxic subjects and that microhemorrhages would be detectable in subjects suffering from clinically apparent HACE during the climb.

## Material and Methods

### Setting

The prospective observational cohort study was performed in the context of the Swiss High Altitude Medical Research Expedition 2013 to Mount Himlung Himal (7126m). Baseline and post-expedition testing was in two groups in Switzerland (550m) eight and nine weeks before the start of the expedition and four and five weeks after return. No supplementary oxygen was used during the climb. Throughout the entire expedition food and fluids were provided in unlimited amounts to the participants.

### Participants

The study included forty healthy subjects aged between 18 and 70 years. Subjects had to be healthy, aged between 18 and 70 years, physically fit and have basic mountaineering experience and skills. Subjects with a history of any neurological, cardiac or respiratory disease, diabetes mellitus type I or II, head trauma or who developed severe AMS, HACE or high altitude pulmonary edema (HAPE) after a rapid ascent (< 3 nights) at altitudes below 3500m and subjects on regular medication with beta-blockers, ACE-inhibitors, nitrates and calcium antagonists, corticosteroids, anti-inflammatory drugs, platelet aggregation inhibitors and anticoagulants were excluded from the study.

### Study size

The number of subjects was determined by the maximum number of climbers that could participate in the expedition when considering the logistical and safety constraints of an ascent to extreme altitudes.

### MR imaging technique

MRI of the brain was performed on a 3 T MR scanner (Magnetom Verio, Siemens, Erlangen, Germany) with a 32-channel head coil during baseline and post-expedition testing. The MRI protocol included 1 mm 3D fluid attenuated inversion recovery (FLAIR,TR/TE 5000/395ms), axial susceptibility-weighted imaging (SWI, slice thickness 1.2mm, TR/TE 28/20ms), and 1mm 3D modified driven equilibrium fourier transform (MDEFT) sequences (TR/TE 7.92/2.48 ms). 1mm multiplanar reformations of FLAIR and MDEFT sequences in axial and coronal planes were made. SWI minimum-intensity projections (MinIP, slice-thickness 9.6mm) were automatically calculated by the scanner. Images were visually assessed for white matter lesions (WML) (FLAIR), signs of cortical atrophy such as widening of sulci (MDEFT), and microhemorrhages (SWI). Number, size, configuration and distribution of WML were recorded. Susceptibility-weighted images were scrutinized for focal hypointense artefacts suggestive of microhemorrhages.

Comparison between pre- and post-expedition MR images was performed in consensus reading by two experienced neuroradiologists blinded to clinical and physiological data (MH, RK). For a more detailed evaluation of possible brain volume changes after altitude exposure, partial volumes of cerebrospinal fluid (CSF), grey matter (GM), and white matter (WM) were estimated on high-resolution T1-weighted images (MDEFT) (software package FSL, version 5.0, Oxford Centre for Functional MRI of the Brain [FMRIB]). All voxels were classified into three tissue classes (CSF, GM and WM) with the sum of probabilities equal to one. Partial volume estimates (PVE) for each tissue class were calculated by integrating the tissue class probabilities voxelwise over the whole volume and expressed as fractions of total brain volume. Physiological changes of PVEs due to aging of subjects between examinations were accounted for by comparison with reference data from adult non-climbers [16, 17].

### Clinical parameters

Subjects baseline characteristics including a detailed medical history and the number of nights spent at an altitude of >4000m at any time before study inclusion was assessed during baseline testing. During the expedition symptoms of AMS were assessed daily using the cerebral score of the environmental symptoms questionnaire score (AMS-c) and the Lake Louise AMS protocol (AMS LL) [18]. An AMS-c ≥0.7 or an AMS LL ≥ 3 plus the presence of headache define occurrence of AMS. Finger pulse oximetry was performed in the morning and evening at rest in a sitting position (Onyx 9500 SportStat, Nonin Medical, Plymouth, USA) after having stable values during at least 3 min. AMS scores and oxygen saturation (SpO_2_) were recorded in a research diary by each subject. Subject groups were accompanied by one or more expedition physicians at all times who monitored the climbers for occurrence of altitude related illnesses and provided appropriate treatment. AMS treatment consisted of single doses of ibuprofen and/or acetaminophen and occurred after AMS scoring. Prophylactic use of analgesics or the use of acetazolamide was not permitted and not supplied to the subjects.

### Statistics

Clinical data is presented as mean and standard deviation (SD) or median and lower and upper limit of the interquartile range (IQR) for parametric and non-parametric data. For comparisons of measurements on different altitudes and group comparisons Kruskal-Wallis test followed by Dunn's Multiple Comparison or Mann-Whitney U-Test was used. One-sample t-test was used to test if mean changes of PVE fractions of different brain tissue classes significantly differed from zero. We assumed a physiological age-related change of CSF, GM and WM fraction of 0.065%, -0.058% and 0.00%, respectively during the 119 day period between baseline and post-expedition testing [16, 17]. Pearson product-moment correlation coefficient with false discovery rate (FDR) correction was computed to assess the relationship between PVE fraction changes and subject characteristics and altitude measurements. For all analyses a p-value of <0.05 was considered statistically significant. Statistical analysis was performed using standard statistical packages (IBM SPSS Statistics Version 20).

*Ethics approval*: The study was approved by the institutional review board of the Canton of Bern, Switzerland. Written informed consent was obtained from all subjects before enrolment.

## Results

### Participant

One subject was excluded from the expedition due to the incidental finding of a brain cavernoma and one subject refused post-expedition imaging due to claustrophobia. A total of 38 subjects, 20 male and 18 female with a mean age of 45.4 years (median 47.5, range 24–69) with a complete set of MRI were included for further analysis.

### Course of the expedition and clinical findings

**Fig 1:**
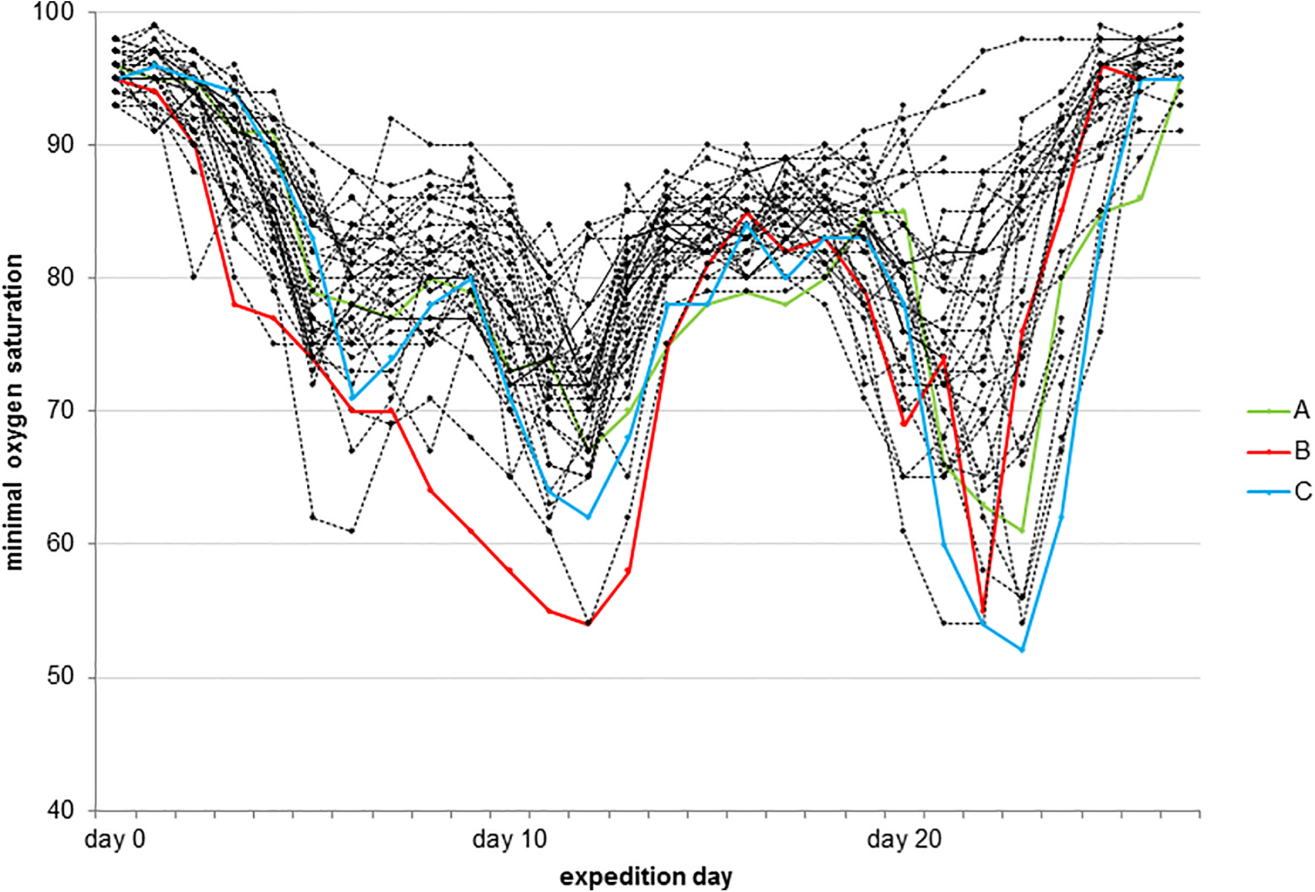
Oxygen saturation of all climbers during the expedition. Oxygen saturation measured by pulse oximetry of all climbers during the expedition. Oxygen saturation measurements of subjects with new findings in post-expedition MRI are indicated in color.

The climbers reached the Himlung basecamp (4800m) on day 5, camp 1 (5465m) on day 10, camp 2 (6025m) on day 11 of the expedition. Camp 3 (7050m) was reached on day 21 and 22 and the summit (7126m) on day 22 and 23 by the two expedition groups. Maximum altitude for overnight stay was camp 1 in 3 subjects, camp 2 in 19 subjects and camp 3 in 15 subjects. The summit was reached by 13 climbers. Detailed subject characteristics and relevant parameters regarding altitudes, AMS scores and oxygen saturation of subjects stratified by occurrence of new MRI findings (described below) is provided in Table 1. Fig 1 shows the results of daily SpO_2_ measurements during the expedition. Table 2 summarizes measurements of oxygen saturation and reported AMS scores at different altitudes between basecamp and camp 3. All individual SpO_2_ measurements, AMS scores and single AMS items collected during the expedition are reported in S1 Table. The main analysis revealed a significant decrease in oxygen saturation with increasing altitude, and concomitant increase of AMS scores and incidence of reported AMS scores above the diagnostic cut-off at higher altitudes compared to BC values. However, post-hoc analysis did not demonstrate a significant difference in oxygen saturation and AMS-c between camp 2 and camp 3 and of AMS LL between basecamp, camp 1 and camp 2 compared to camp 3. No significant change in body weight occurred when comparing measurements before and after altitude stay (p = 0.371).

**Table 1:** Subject characteristics and relevant parameters regarding altitudes, AMS scores and oxygen saturation.

Subject ID	Age (years)	Gender	Maximal sleep altitude (m)	Maximal altitude reached (m)	Lowest SpO_2_ (%)	Number of days SpO_2_<75%	Maximal AMS-c	Number of days AMS-c >0.7	Maximal AMS LL	Number of days AMS LL >2	highest altitude reached during summit attempt; reason for aborting climb
1	32	f	6025	6025	65	5	0.957	1	8	15	6025m; exhaustion
2	30	m	7050	7126	61	4	1.078	1	6	8	summit
3	53	m	7050	7126	56	8	0.179	0	4	10	summit
4	54	m	6025	6025	71	2	0.269	0	4	4	6025m; AMS, exhaustion
5	56	f	6025	6025	64	3	0.497	0	7	7	no summit attempt due to severe exhaustion at 6025 during acclimatisation period
6	32	f	6025	6900	66	6	0.418	0	5	6	6900m, hypothermia, exhaustion
7	36	m	5465	5800	71	8	2.895	3	9	8	5800m, mild AMS, exhaustion
8	47	m	7050	7126	65	8	1.755	1	2	0	summit
9	43	f	7050	7050	66	2	0.349	0	0	0	6900m, dyspnoea, possible HAPE
10	31	f	7050	7126	56	6	2.626	2	10	10	summit
11	27	f	7050	7126	67	4	0.298	0	0	0	summit
12	63	m	6025	6350	62	13	0.643	0	7	13	6360m, mild to moderate AMS
13	58	m	7050	7126	65	5	0.090	0	3	2	summit
14	48	f	6025	6025	81	0	0.874	1	6	6	no summit attempt due to AMS and exhaustion at 6025 during acclimatisation period
15	62	m	6025	6025	72	1	0.273	0	3	2	5600m; dyspnoea and exhaustion, subsequent clinical and radiologic finding of central pulmonary embolism
16	48	m	6025	6250	67	5	1.508	4	9	10	6250m; moderate AMS
17	57	f	6025	6025	72	5	1.090	2	6	7	6000m; metatarsal fracture during summit attempt
18	25	f	7050	7126	54	6	0.855	1	5	5	summit
19	58	f	7050	7126	62	0	0.333	0	3	2	summit
20	42	f	6025	6900	54	6	0.698	0	4	4	6900m, frost bite and hypothermia
21	47	m	7050	7126	65	6	0.608	0	6	8	summit
22	57	m	7050	7126	65	4	1.271	1	8	7	summit
23	52	f	6025	6025	72	1	0.343	0	5	7	5600m, exhaustion
24	26	f	5465	5600	73	3	0.267	0	6	2	5600m; exhaustion, lack of motivation
25	53	f	6025	6025	70	2	0.411	0	8	6	no summit attempt, due to cardiac arrhythmia during acclimatisation period
26	62	m	7050	7126	73	1	0.569	0	3	2	summit
27	36	f	6025	6025	65	4	1.112	1	8	12	6025m; exhaustion, fear of difficult conditions
28	48	m	6025	6900	71	1	0.700	0	7	9	6900m; accompanied descent of partner with frostbite and hypothermia
29	37	f	6025	6025	72	1	0.564	0	6	5	no summit attempt, cardiac arrhythmia during acclimatisation period
30	35	f	6025	6025	54	9	1.011	2	9	9	5600;m exhaustion, lack of motivation
31	50	m	6025	6025	65	5	1.370	2	7	11	6025m; moderate AMS
32	44	f	6025	6025	67	4	0.244	0	12	9	5400m; exhaustion, moderate AMS
33	69	m	6025	6300	67	5	0.154	0	3	2	6300m; hypothermia
34	54	m	6025	6450	74	1	0.000	0	0	0	6025m; dyspnoea, cough, possible HAPE
35	41	m	5465	5600	77	0	1.023	2	9	12	5600; exhaustion, lack of motivation
A	24	m	7050	7126	61	7	1.478	3	12	9	summit
B	32	m	7050	7050	54	12	0.766	1	4	2	7050m; exhaustion
C	56	m	7050	7050	52	10	0.386	0	3	4	7050m; severe dyspnoea, possible HAPE

AMS-c, cerebral score of the environmental symptoms questionnaire score; AMS-LL, acute mountain sickness according to Lake Louise AMS protocol; BC, basecamp, C1–3; camps 1–3; IQR, interquartile range; SpO_2_, pulse oximetric arterial oxygen saturation.

**Table 2:** Oxygen saturation and reported AMS scores at different altitudes.

	BC (4800m)	C1 (5650m)	C2 (6025m)	C3 (7050m)	significance
oxygen saturation (%)	84 (IQR 81–87)	79 (IQR 75–82)	72 (IQR 69–76)	65 (IQR 59–67)	p < 0.0001
AMS-c	0.0 (IQR 0–0)	0.0 (IQR 0–0.09)	0.09 (IQR 0–0.40)	0.34 (IQR 0.12–0.62)	p < 0.0001
% AMS-c ≥0.7	2.6	1.8	12.7	21.4	p < 0.0001
AMS LL	0.0 (IQR 0–0)	0.0 (IQR 0–0)	0.0 (IQR 0–3)	0.0 (IQR 0–1.5)	p < 0.0001
% AMS LL ≥3	7.0	10.6	28.2	21.4	p < 0.0001

AMS-c, cerebral score of the environmental symptoms questionnaire score; AMS-LL, acute mountain sickness according to Lake Louise AMS protocol; BC, basecamp, C1–3; camps 1–3; IQR, interquartile range; SpO_2_, pulse oximetric arterial oxygen saturation.

### Imaging findings

Image quality was considered excellent throughout all sequences. On baseline MRI 16 subjects had no WML. In 22 subjects small focal WML were observed, the number ranging from 1–10 (n = 14), 11–20 (n = 5), and 20–30 lesions (n = 3). All of these lesions were small, most ≤ 1mm, not confluent, mostly located in the subcortex or deep white matter, and considered to be of no clinical importance. There was a significant correlation between subject age but not number of nights spent above 4000m previous to study inclusion and number of WML at baseline (R square 0.153, p = 0.015 and R square 0.0002, p = 0.9285, respectively). On follow-up imaging the number of WML remained unchanged in all 38 subjects, no new lesions were observed.

**Fig 2:**
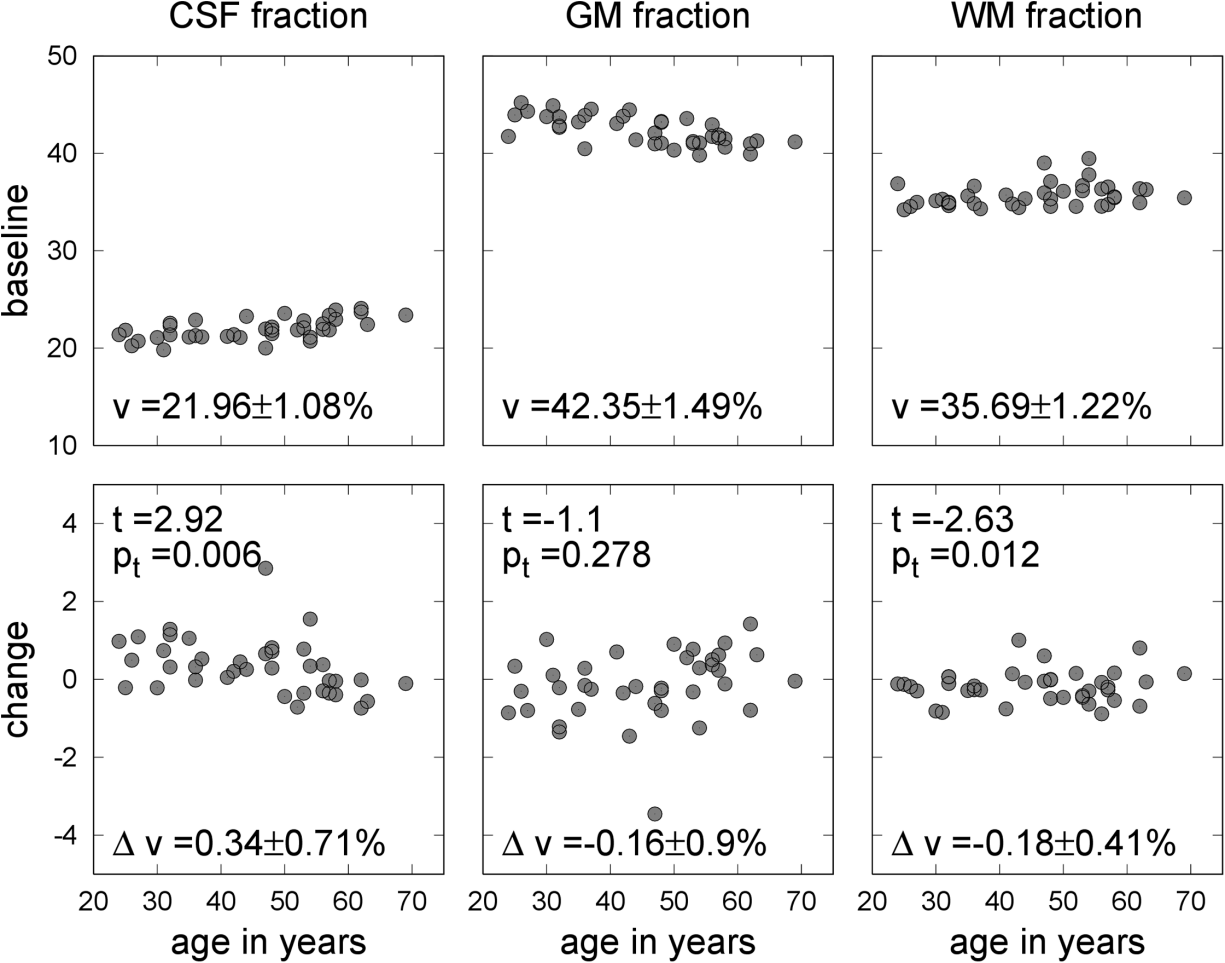
Partial volume estimate fractions at baseline and changes between baseline and post-expedition imaging. Top: Age dependence of the percentages of the partial volume estimates for cerebro-spinal fluid, grey matter and white matter during baseline testing. Means and standard deviations are reported at the bottom of the panels. Bottom: Partial volume estimates fraction change between baseline and post-expedition imagining as a function of age. Means and standard deviations are reported at the bottom of the panels. At the top of the panels the results of a one-sample t-test of the hypothesis that the mean PVE change is zero are given.

Fig 2 shows the distribution of the PVE fractions at baseline and PVE fraction changes between baseline and post-expedition testing. Compared to baseline testing, we observed a significant mean CSF fraction change (0.34% [95% CI 0.10–0.58], p = 0.006) and a significant mean WM fraction change (-0.18% [95% CI -0.32–-0.04], p = 0.012), whereas the mean GM fraction remained stable (mean change -0.16% [95% CI -0.46–0.13], p = 0.278). The expected physiological mean PVE changes due to aging were outside of the observed lower and upper 95% CI limits for CSF and WM fraction changes. After correction for multiple comparisons, no significant correlations between PVE fraction changes and subject characteristics, body weight changes or parameters determined by severity of hypoxia were observed.

**Fig 3:**
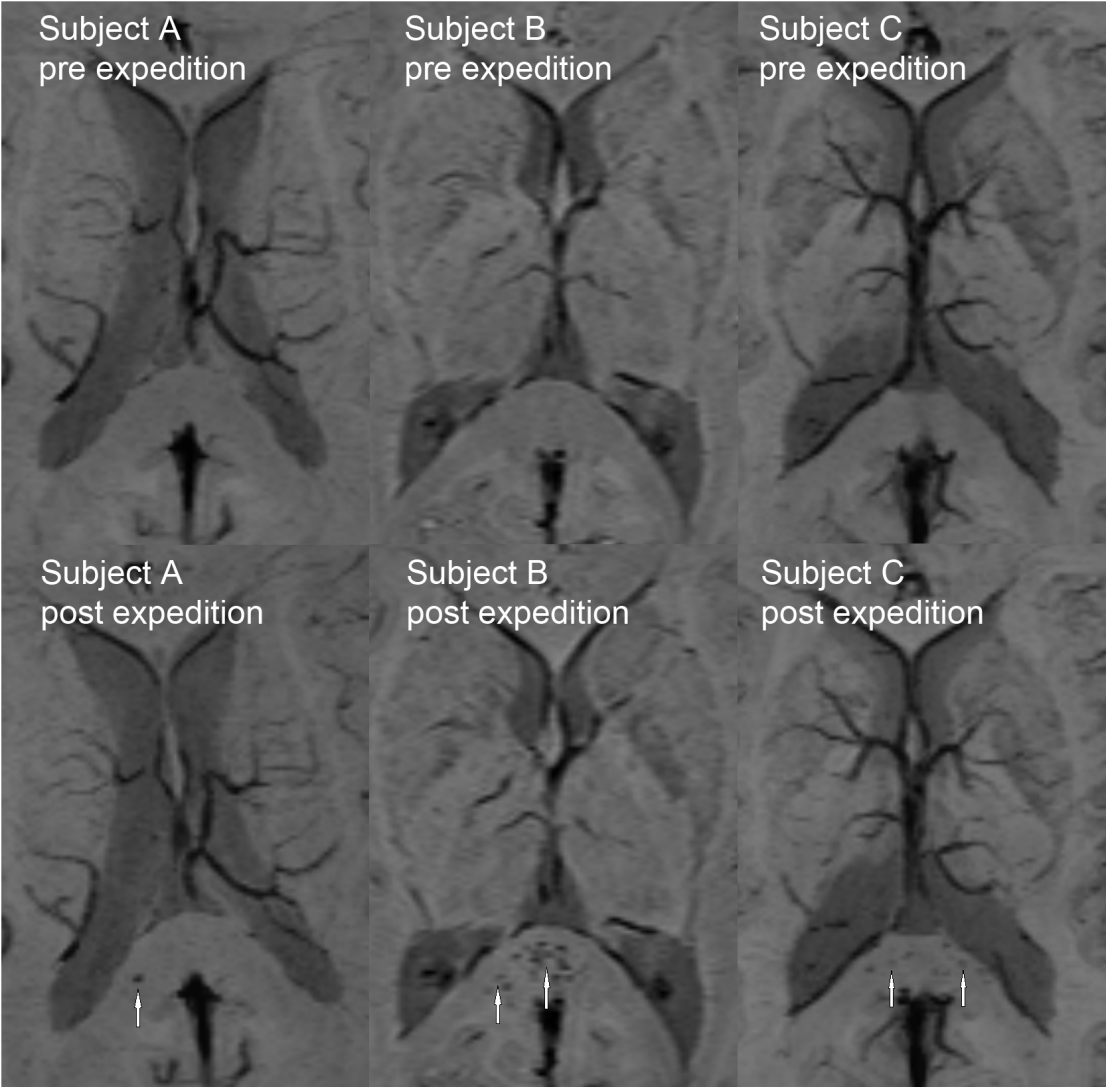
Imaging findings pre- and post-expedition in subjects with new microhemorrhages after the climb. Subject A: Comparison of SWI (MinIP) in pre- and post-expedition MRI shows a new solitary microhemorrhage (indicated by arrow). SWI of Subject B and SWI (MinIP) of subject C show multiple new microhemorrhages of the corpus callosum visible in post-expedition MRI (arrows).

On post-expedition MRI one subject (subject A) presented a new solitary hypointense splenial lesion with the same imaging characteristics described above, compatible with a micro hemorrhage. Two subjects showed multiple new focal hypointensities in the splenium of the corpus callosum, not apparent on T1- and T2-weighted images, on post-expedition imaging suggestive of multiple microhemorrhages. Subject B also showed faint new signal hypointensities in the corpus and genu of the corpus callosum. No microbleeds in other regions of the brain were detected (Fig 3). Subjects with new microhemorrhages after the expedition had a significantly higher maximal sleep altitude (p = 0.048), and significantly lower minimal recorded SpO_2_ values (p = 0.007), and a higher number of days with reported SpO_2_ values below 75% than subjects with unaltered MRI (p = 0.007). Maximal AMS-c and AMS LL scores and total number of days with AMS scores above the diagnostic cut-off did not differ between subjects with and without new microhemorrhages (Table 3).

**Table 3:** Comparison of subjects with and without new MRI findings.

	Age (years)	Gender	Maximal sleeping altitude (m)	Maximal altitude reached (m)	Lowest SaO_2_ (%)	Number of days SpO_2_<75%	Maximal AMS-c	Number of days AMS-c >0.7	Maximal AMS LL	Number of days AMS LL >2
Significance	0.297	0.152	0.048	0.168	0.007	0.007	0.959	0.405	0.959	0.644

AMS-c, cerebral score of the environmental symptoms questionnaire score; AMS-LL, acute mountain sickness according to Lake Louise AMS protocol; SpO_2_, pulse oximetric arterial oxygen saturation.

## Discussion

The present study evaluating the occurrence of structural cerebral changes in a cohort of 38 recreational high altitude climbers has three main findings. We did not detect structural changes indicating brain damage due to chronic hypoxia at altitude, such as WM hyperintensities or global cortical atrophy. We documented a small but significant decrease in global WM volume after prolonged hypobaric hypoxia. Post-expedition MRI revealed one subject with a solitary microhemorrhage and two subjects with multiple new microhemorrhages in the splenium of the corpus callosum. None of these subjects had experienced clinical symptoms of cerebral dysfunction indicative of HACE during altitude exposure. All three climbers had spent a night at an altitude above 7000m and reported significantly lower minimal SpO_2_ values, more days with SpO_2_ values lower than 75% but not higher AMS scores than climbers with unaltered post-expedition MRI.

Our study has a number of limitations. Less than 45% of climbers reached an altitude of more than 7000 m. The majority of subjects only attained altitudes between 5645 m and 6900 m for various reasons including altitude illnesses, exhaustion and injuries. The applied AMS scoring systems for identifying the presence and severity of AMS depend on the subjective rating of the severity of symptoms by the afflicted person. Despite their widespread use it has been disputed whether these scoring systems can accurately diagnose AMS [14, 15] and studies comparing different scoring systems revealed differences in scoring results [19]. Additionally, AMS susceptibility is influenced by age which may have influenced AMS incidence in our cohort with a large age range. We did not perform serial post-expedition MRI. We therefore cannot exclude that WM or GM changes had already resolved before or occurred after post-expedition MRI, nor can we determine if the observed reduction in WM fraction is reversible in the long-term. Our findings are based on the comparison of MRI before and after a single episode of hypobaric hypoxia and cannot be generalized to repetitive exposure. Our results are generalizable to the general population of non-elite, recreational high altitude climbers and trekkers participating in sojourns that include camps not higher than 7000 m and without the use of oxygen. We assume that the generalizability of our findings can be extended to include subjects climbing at higher altitudes, provided that supplementary oxygen is used to avoid more severe hypoxic conditions.

Only a few studies have examined the possible effect of severe hypobaric hypoxia on cerebral structural integrity. In two studies with a transversal design (comparison to a matched group of non-climbers), Garrido et al. described findings of cortical atrophy and periventricular T2-hyperintensities in groups of 26 and 21 mountain climbers ascending to altitudes of 7000 m and 8000 m [8, 10]. Fayed et al. reported results of MRI in 35 climbers after ascents to peaks of different heights ranging from Mont Blanc (4810 m) to Mt. Everest (8848 m). In the majority of these climbers, including in three climbers after ascent to the relatively moderate altitude of Mont Blanc, occurrence of subcortical lesions and cortical atrophy were reported after visual evaluation of 5 mm FLAIR and T2-weighted images when compared with non-climbing controls [7]. Two studies applying a longitudinal design with MRI before and after high altitude exposure have reported structural cerebral alterations. In 2008 Di Paola et al. found reduced grey matter density/volume in the left angular gyrus but no WM lesions in 9 professional climbers 8 weeks after ascents to K2 and Mt Everest using voxel-based morphometry on magnetization prepared rapid acquisition gradient echo (MPRAGE), and on T2 and FLAIR sequences [20]. Garrido et al. reported new high-intensity lesions on T2-weighted images in the posterior lobes in 2 of 9 climbers after reaching altitudes between 7800 m and 8463 m [9]. Anooshiravani et al. did not detect WM lesions or cortical atrophy in a group of 8 climbers when comparing MRI before and after an ascent to altitudes of up to 7100 m [21]. Interestingly, subcortical WM lesions have also been reported after repeated occupational exposure to nonhypoxic hypobaria [22]. This indicates that the low ambient pressure at extreme altitudes per se might be a factor contributing to the development of WM lesions described in previous studies.

In contrast to T2 and FLAIR images with slice thicknesses of 4–6 mm at MR Field strengths of 0.5–1.5 Tesla in previous studies, we performed an isotropic 3D FLAIR sequence at 3 Tesla which is highly sensitive for focal WML. The study at hand examined the largest subject group included in a longitudinal imaging study so far, even when only considering the subjects who reached an altitude of more than 7000m. In our subjects, we did not detect evidence for grey matter atrophy as reported in previous studies. One possible explanation could be that our subjects only reached an altitude of 7126 m compared to the higher altitudes attained by Garridos and Di Paolas groups [5, 9]

A significant decrease in brain WM fraction, even after correction for physiological aging-associated changes, was documented in our subjects on post-expedition imaging. This finding has not yet been reported in the context of prolonged hypobaric hypoxia. White matter alterations have been demonstrated in MRI of patients suffering from obstructive sleep apnea (OSA). These findings include impairment of fiber integrity throughout the brain but with more pronounced changes in the cortices, limbic system, pons, and cerebellum tracts [23, 24]. In OSA repeated airway obstructions lead to intermittent nocturnal hypoxic episodes. Similarly, at high altitudes, nocturnal central apnea in the context of periodic breathing leads to pronounced oxygen desaturation periods [25, 26]. Repeated deoxygenation with apneic episodes may lead to a number of oxidative and inflammatory processes in OSA patients and healthy subjects at high altitudes. In both groups, markers of oxidative stress and inflammation are elevated [25–27] which have been associated with neuronal and glial injury [28]. Furthermore, nutritional factors could potentially contribute to the observed decrease in WM fraction. Decreases in global WM and GM volumes have been reported in patients suffering from anorexia nervosa [29, 30] and decreases in GM volumes in subjects participating in a multi-day ultra-marathon [31]. In both groups WM and GM returned to normal after restoration of body weight. Lack of appetite and weight loss is common in high altitude climbers [32], but the duration and extent of body mass reduction is far less severe than in anorexia patients.

Reversible vasogenic edema and microhemorrhages indicating blood-brain barrier disruption with a predilection for the splenium of the corpus callosum are reported to be a finding characteristic of HACE [6, 11]. Kallenberg et al., examined 6 subjects after the occurrence of AMS and non-fatal HACE and detected microhemorrhages in the corpus callosum in the 3 subjects who had suffered from HACE, but not in the 3 subjects who only had experienced severe AMS [6]. The same group postulated a high diagnostic specificity for HACE of microhemorrhages in the corpus callosum in a later cross-sectional study comparing 37 mountaineers that had either experienced HACE, HAPE, severe AMS or no high altitude illness [12].

Three of our climbers showed new microhemorrhages in the splenium of the corpus callosum after the climb. Microhemorrhages represent evidence for substantial disruption of the blood brain barrier which is the most important contributor for development of HACE [33], a condition associated with a high mortality especially at extreme altitudes where treatment possibilities are limited. We have to assume that these climbers were at an increased risk for development of clinically apparent brain edema. However, none of them demonstrated clinical symptoms of HACE or severe AMS during the course of the expedition. The lack of reported symptoms demonstrates that a substantial disruption of the blood-brain barrier can occur in severe hypoxia without obvious clinical symptoms being detected in the challenging circumstances at extreme altitude.

In conclusion, we cannot confirm that a sojourn to extreme altitudes is associated with the development of focal WM lesions and GM atrophy indicative of long-term cerebral damage in a majority of climbers. High altitude climbing leads to a decrease in brain WM fraction, possibly caused by the associated prolonged hypoxic and catabolic state. Furthermore, microhemorrhages indicative of substantial blood-brain barrier disruption occur in a significant number of climbers attaining extreme altitudes. In climbers with blood-brain barrier disruption, clinical symptoms of brain edema can be absent or are missed by the affected climber and their fellow mountaineers in a state of extreme exhaustion and mental fatigue which is characteristic of high altitude climbing.

## Supporting Information

S1 Table Oxygen saturation, AMS scores and single AMS items as collected during the expedition. (XLSX)
